# Inhibition of Growth of Colon Tumors and Proliferation of HT-29 Cells by *Warburgia ugandensis* Extract through Mediating G_0_/G_1_ Cell Cycle Arrest, Cell Apoptosis, and Intracellular ROS Generation

**DOI:** 10.1155/2021/8807676

**Published:** 2021-12-29

**Authors:** Yongli Zhang, Guilin Chen, Xiaocui Zhuang, Mingquan Guo

**Affiliations:** ^1^CAS Key Laboratory of Plant Germplasm Enhancement and Specialty Agriculture, Wuhan Botanical Garden, Chinese Academy of Sciences, Wuhan 430074, China; ^2^The Sino-Africa Joint Research Center, Chinese Academy of Sciences, Wuhan 430074, China; ^3^Innovation Academy for Drug Discovery and Development, Chinese Academy of Sciences, Shanghai 201203, China; ^4^University of Chinese Academy of Sciences, Beijing 100049, China; ^5^School of Chemical Biology and Environment, Yuxi Normal University, Yuxi 653100, China

## Abstract

*Warburgia ugandensis* Sprague (*W. ugandensis*), widely distributed in Africa, is a traditional medicinal plant used for the treatment of various diseases including cancer. We intended to evaluate the anticolorectal cancer (CRC) activities of the crude extract from *W. ugandensis* (WUD) and reveal the underlying molecular mechanisms of its action. We found that WUD inhibited the proliferation of HT-29 and HCT116 cells in a time- and dose-dependent manner and induced intracellular ROS generation. The inhibitory effect of WUD on the proliferation of HT-29 and HCT116 cells could be attenuated by NAC (a ROS scavenger) in a dose-dependent manner. WUD induced G_0_/G_1_ phase arrest, down-regulated the protein expression of Cyclin D1 via ROS accumulation in HT-29 cells. In search of the molecular mechanism involved in WUD-induced Cyclin D1 down-regulation, it was found that WUD can suppress PI3K/Akt/GSK3*β* signaling pathway in HT-29 cells. Next, it was found that WUD also activated apoptosis, poly-ADP ribose polymerase 1 (PARP1) cleavage and down-regulated pro-caspase 3 in HT-29 and HCT116 cells. Besides, WUD decreased the growth of colon tumors *in vivo* in the xenograft mouse model. We demonstrated for the first time that ROS and their modulation in the corresponding intracellular signaling could play a significant role in the potential activity of WUD against CRC cells.

## 1. Introduction

Cancer is the second leading cause of death worldwide, resulting in an estimated 10.0 million deaths in 2020. The most commonly diagnosed cancers are breast cancer, lung cancer, colorectal cancer (CRC), prostate cancer, and stomach cancer. Among them, CRC ranks third in morbidity (10.0% of the total cases) and second in mortality (9.4% of the total cancer deaths) [1]. It is noteworthy that the CRC incidence of younger individuals is increasing [2].

Current standard treatments for CRC mainly include surgery, chemotherapy, radiotherapy, immunotherapy, and targeted therapy. Due to the absence of typical symptoms in the early stage of CRC, many patients with CRC are detected by symptoms or signs that usually appear in the advanced stages, with an extremely low 5-year survival rate (14%) compared to initial stages (90%) [3]. The facts above make chemotherapy the primary choice, which is limited by side effects, undesirable toxicity, and drug resistance. Therefore, it is urgent to develop more effective but low-toxicity anti-CRC drugs.

*W. ugandensis*, which belongs to the Canellaceae family, is widely used in traditional medicine within Eastern and Southern Africa for the treatment of various diseases and also as a dietary additive [4]. Traditional medicine practitioners in Africa used *W. ugandensis* to treat malaria, tuberculosis, tapeworm, trypanosomiasis, leishmaniasis, and candida albicans infection [5–8]. Besides, *W. ugandensis* is also used for HIV infection and measles virus infection [9]. In addition to the above-mentioned antibacterial, antifungal, and antiviral effects, *W. ugandensis* also possesses potent anticancer activity. According to an ethnobotanical study, *W. ugandensis* was used to treat breast cancer, cervical cancer, prostate cancer, skin cancer, throat cancer and intestinal cancer in the rural Greater Mpigi region of Uganda [10]. In addition, a number of studies have documented the cytotoxicity of some compounds in *W. ugandensis* or in other plant extracts against different types of cancer, including mouth epidermoid carcinoma [5, 11, 12], ovarian cancer [13], breast cancer [14, 15], prostate cancer [16], melanoma [14, 17] and glioblastoma [14]. Our previous study showed that WUD exhibited potent inhibitory growth activity against HT-29 CRC cells. However, the anti-CRC molecular mechanisms of *W. ugandensis* have not been revealed. Besides, as a dietary medicinal plant, the leaves, stem barks, roots, and fruits of *W. ugandensis* are used in the daily diet, which suggests that it is safe to humans [9].

Apoptosis is a well-characterized type of programmed cell death (PCD), and plays a critical role in maintaining homeostasis by removing unnecessary and dysfunctional cells. It is mediated through the intrinsic pathway and extrinsic pathway [18].

A fundamental feature of cancer is the deregulation of cell cycle progression, which is positively controlled by cyclins and cyclin-dependent kinases (CDKs). Cyclins function as the regulatory partners of cyclins/CDK complex. The Cyclin D1 regulates the G_1_/S progression by binding to CDK4 and CDK6 [19]. Cyclin D1 is upregulated in various cancers including at least one-third CRC and contributes to the development and progression of CRC [20]. In addition, Cyclin D1 was also considered an unfavorable prognostic factor of CRC [21]. The over-expression of cyclin D1 in cancer cells is frequently resulting from the deregulation of ubiquitin-dependent proteasomal degradation system. The degradation of Cyclin D1 is tightly regulated by multiple kinases, including glycogen synthase kinase 3-*β* (GSK3*β*), which phosphorylates Cyclin D1 at threonine 286 (T286), and thus triggers the CRM1-mediated nuclear export and the following ubiquitin-dependent proteasomal degradation of Cyclin D1 within the cytoplasm [22]. In these cell signaling cascades, GSK3*β* is negatively regulated by the Akt signaling pathway. Akt participates in the regulation of cell cycle progression by the phosphorylation at Serine 9 of GSK3*β*, which leads to the inhibition of GSK3*β* activity [19]. The PI3K/Akt pathway is one of the most important pathways related to tumor progression by stimulating cell survival and suppressing apoptosis. The PI3K/Akt pathway is frequently activated in human cancers, including CRC [23].

Redox-homeostasis is essential for diverse cellular processes of normal and cancer cells. Reactive oxygen species (ROS) are a group of short-lived, reactive, unstable, partially reduced oxygen derivatives of oxidative phosphorylation, mainly produced in mitochondria. ROS include superoxide (O_2_^−^, the precursor of most of the ROS), hydrogen peroxide (H_2_O_2_), hydroxyl radical (^∙^OH), singlet oxygen (^1^O_2_), and hypochlorous acid (HOCl) [24]. In cancer cells, several factors including the increased metabolism, dysfunction of mitochondria, up-regulation of a number of well-known oncogenes (*c-myc*, *k-ras*, and *BRAC1*), hypoxia, and signaling associated with ROS production may promote the generation of ROS [24, 25]. Many chemotherapeutic agents such as motexafin gadolinium, doxorubicin, cisplatin, 2-methoxyestradiol, buthionine sulfoximine, imexon, isoorientin, curcumin, and triterpenoids, including betulinic acid, celastrol, and the methyl ester of 2-cyano-3,12-dioxo-oleana-1,9-dien28-oic acid (CDDO-Me), exert anticancer effect via inducing high levels of cellular ROS, which can disrupt the structure of DNA, protein and lipids and eventually induce cell death [26–28].

In this study, we investigated the ROS-dependent growth inhibitory, cell cycle arrest, and apoptotic effects of WUD on HT-29 and HCT116 cells and managed to elucidate the molecular mechanisms underlying its potential anti-CRC activity.

## 2. Materials and Methods

### 2.1. Chemicals and Reagents

McCoy's 5A medium, RPMI-1640, 100 U/mL streptomycin, 100 *μ*g/mL penicillin and PBS were acquired from gibco (Life Technologies, USA). Fetal bovine serum was obtained from BI (Bioind, Israel). SRB assay kit was obtained from BestBio (Shanghai, China). Cell cycle and apoptosis analysis kits were purchased from Yeasen Biotech (Shanghai, China). Annexin V-FITC/PI apoptosis kit was obtained from Multisciences (Hangzhou, China). Reactive Oxygen Species Assay Kit was obtained from Beyotime (Shanghai, China). Anti-PARP1, anti-caspase 3, anti-GAPDH, anti-GSK3*β*, anti-p-GSK3*β* (Ser9), anti-Cyclin D1, anti-*β*-actin, anti-PI3K, anti-Akt, and anti-p-Akt (Ser473) antibodies were purchased from ProteinTech (Wuhan, China). Anti-cleaved caspase 3 was purchased from Cell Signaling Technology (Beverly, MA, USA). Ultrapure water was produced by the ultrapure water system (Yipu Yida Technology, Nanjing, China). Cisplatin was purchased from Acmec (Shanghai, China). All the analytically pure chemicals and solvents were purchased from Sinopharm Group (Shanghai, China).

### 2.2. Plant Material

The root barks of *W. ugandensis* were collected in August 2015 from Mountain Kenya, Kenya. The plant was kindly authenticated by Prof. Guangwan Hu, a senior taxonomist of Wuhan Botanical Garden, Chinese Academy of Sciences. A voucher specimen (No. 2015001) was deposited in the herbarium of the Key Laboratory of Plant Germplasm Enhancement and Specialty Agriculture of Chinese Academy of Sciences.

### 2.3. Sample Preparation

Air-dried *W. ugandensis* root barks were grinded into fine powder, and 5.0 g samples were taken, and extracted 3 times (12 h each time) at room temperature with 50.0 mL dimethyl carbonate (DMC). The obtained extract solution was filtered and evaporated under vacuum to afford the crude extract, and the DMC extract was named as WUD for a further study in this work.

### 2.4. Cell Culture and Cell Proliferation Assay

HT-29 cell line, HCT116 cell line, and CT26 cell line were maintained in McCoy's 5A (HT-29 and HCT116) or RPMI-1640 (CT26) medium supplemented with 10% fetal bovine serum (FBS), 100 U/mL streptomycin, and 100 *μ*g/mL penicillin in a cell incubator under humidified conditions with 5% CO_2_ at 37°C. To evaluate the anti-proliferate activity of WUD against HT-29 and HCT116 cells, the SRB assay was implemented according to the manufacturer's instructions (Beyotime, Shanghai, China). In brief, cells were seeded into 96-well plates at a density of 1.0 × 10^4^ per well. After 24 h incubation, WUD was diluted with cell culture medium and added to each well at the indicated concentrations. Cell viability was assessed by SRB. Absorbance at a wavelength of 540 nm was measured by a Tecan microplate reader (Tecan Infinite M200 Pro, Tecan Group Ltd., Männedorf, Switzerland).

### 2.5. Cell Cycle Analysis

The cell cycle analysis was conducted using a cell cycle and apoptosis kit (Yeasen Biotech, Shanghai, China) by flow cytometry. In brief, 6.0 × 10^5^ cells per well were seeded in 6-well tissue culture plates and incubated overnight. Cell culture medium was carefully removed and replaced with fresh culture medium containing WUD at different concentrations. After 24 h incubation, cells were trypsinized and collected, washed with ice-cold PBS buffer, fixed with 70% ethanol at -20°C overnight. After fixation, the cells were washed with PBS and stained with propidium iodide (PI) and RNase A for 30 min in the dark at 37°C. At last, the cells were analyzed with flow cytometry (BD Accuri C6, USA).

### 2.6. AnnexinV-PI Assay of Apoptosis

Apoptosis was measured with an Annexin V-FITC apoptosis detection kit (Multisciences, Shanghai, China) according to the manufacturer's instructions. Briefly, 6.0 × 10^5^ cells per well were plated in 6-well tissue culture plates and incubated overnight. Cell culture medium was removed carefully and replaced with fresh culture medium containing WUD at different concentrations. After 24 h incubation, cells were trypsinized and collected. Then, the cell pellets were washed with pre-cold PBS twice and stained with Annexin V-FITC and PI for 5 min at room temperature in the dark. Then, the samples were analyzed with confocal microscopy (Leica DMi8, Wetzlar, Germany) and flow cytometry (BD Accuri C6, USA).

### 2.7. Protein Extraction and Western Blotting

Cells were harvested and rinsed twice with ice-cold PBS and lysed with RIPA cell lysis buffer (Beyotime, Shanghai, China). The protein concentration was quantified by a BCA protein assay kit (Beyotime, Shanghai, China). Cell lysates containing equal amounts of protein were mixed with 2 × Laemmli buffer (Beyotime, Shanghai, China) and resolved by sodium dodecyl sulfate polyacrylamide gel electrophoresis (SDS-PAGE), and then transferred to nitrocellulose membranes (Merck Millipore, Germany) for immunoblotting. After blocked in 0.5% nonfat dry milk, the nitrocellulose membranes were incubated with the indicated primary antibodies at room temperature for 2 h. After incubation with horseradish peroxidase- (HRP-) conjugated secondary antibodies (Beyotime, Shanghai, China), the Immunoreactive signals were visualized using the ECL chemiluminescence system (Merck Millipore, Germany).

### 2.8. Measurement of ROS Generation

ROS determination was performed by using a reactive oxygen species (ROS) assay kit (Beyotime, Shanghai, China) according to the manufacturer's instructions. Briefly, cells were seeded into 96-well plates (1.0 × 10^4^ per well) and incubated for 24 h for adhesion. Then, the cells were treated with varying concentrations of WUD for 24 h. Afterwards, the cells were incubated with DCFH-DA for 20 min in the dark. The DCF fluorescent signals were analyzed by a Tecan microplate reader (Tecan Infinite M200 Pro, Tecan Group Ltd., Männedorf, Switzerland) and a cell imaging multimode reader (Cytation 1, BioTek, USA).

### 2.9. In Vivo Animal Study

5-week-old female Balb/c mice were obtained from Beijing Vital River Laboratory Animal Technology Co. Ltd. (NO. 110011211100400823). All experimental procedures were performed according to the National Institutes of Health Guide for Care and Use of Laboratory Animals and were compliance with Animal Welfare of the National Institutes of Health. For tumor xenograft assay, CT26 CRC cells (0.15 *μ*L, 1.0 × 10^7^/mL) were subcutaneously injected into the right axilla of Balb/c mice. When tumor volume had reached about 70 mm^3^, the mice were randomly divided into 5 groups as follows: (1) model group: administered PBS (200 *μ*L/20 g/d); (2) DDP group: intraperitoneally injected with cisplatin (5 mg/kg, twice a week); (3) WUD-L group: intraperitoneally injected with WUD (57 mg/kg/d); (4) WUD-M group: intraperitoneally injected with WUD (114 mg/kg/d); and (5) WUD-H group—intraperitoneally injected with WUD (228 mg/kg/d). Mouse body weights and tumor volumes were measured three times per week. Tumor volume was calculated as length × width^2^ × 0.5. After 18 days of treatment, the mice were sacrificed, and the tumors were subsequently excised, photographed, and weighed.

### 2.10. Statistical Analysis

Data are presented as the mean ± standard deviation (SD). Differences between groups were assessed using an unpaired two-tailed Student's *t*-test. *P* values < 0.05 were considered significant.

## 3. Results

### 3.1. WUD Inhibits the Proliferation of CRC Cells in a Dose- and Time-Dependent Manner

**Figure 1:**
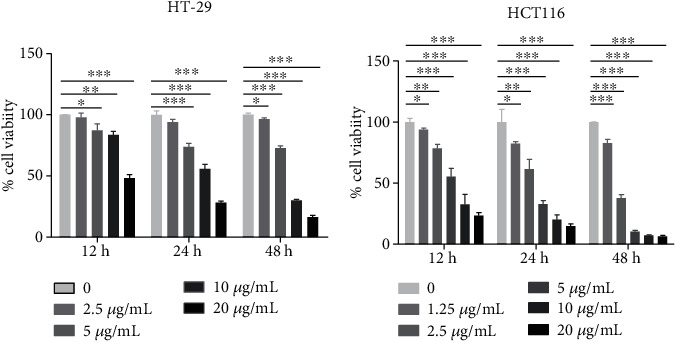
WUD inhibited HT-29 and HCT116 proliferation. HT-29 cells (a) or HCT116 cells (b) were treated with WUD for 12, 24, and 48 h, respectively. Cell viability was determined by SRB assay. ^∗^*P* < 0.05;  ^∗∗^*P* < 0.01;  ^∗∗∗^*P* < 0.001.

To evaluate the kinetics of WUD-induced antiproliferation effects, we treated HT-29 cells and HCT116 cells with various concentrations of WUD at the indicated time points and measured the cell viability via SRB assay. As shown in Figure 1, with the increase of concentration and incubation time, the inhibition effects were more pronounced. The cell viability of HT-29 cells and HCT116 cells was reduced to 16.45% and 6.38% under the treatment of 20 *μ*g/mL WUD for 48 h, respectively. These results indicated that WUD could depress the cell viability of CRC cells in a dose- and time-dependent manner.

### 3.2. WUD Prompts Cellular ROS Generation in CRC Cells

**Figure 2:**
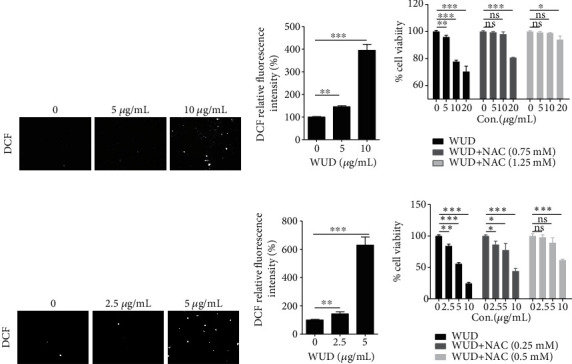
Effect of WUD on ROS production of CRC cells. HT-29 cells (a, b) and HCT116 cells (d, e) were treated with WUD for 24 h; then, the cells were stained with DCFH-DA. The fluorescent intensity of DCF was detected by a microplate reader (a, d) and a cell imaging multi-mode reader (b, e). The fluorescence values of DCF were normalized with the SRB values of the same samples. HT-29 cells (c) and HCT116 cells (f) were pre-treated with NAC for 1 h and then treated with WUD for 24 h. Cell growth inhibition ratio was determined by SRB assay. Data were mean ± SD. ^∗^*P* < 0.05;  ^∗∗^*P* < 0.01;  ^∗∗∗^*P* < 0.001. ns: not significant.

Numerous evidences indicate that cancer cells usually exhibit higher basal levels of ROS, which may implicate in oxidative damage, and cause prominent damage to affected DNA and proteins and further lead to cell death [29]. Our previous study demonstrated that the WUD-induced ROS was involved in its anticancer activity against lung cancer A549 cells. To investigate the effect of WUD on ROS generation in CRC cells, HT-29 cells and HCT116 cells were treated with indicated concentrations of WUD and stained with DCFH-DA, an oxidant-sensitive fluorescent probe, the fluorescence was determined by a microplate reader and a cell imaging mult-imode reader. We found that the intracellular ROS production was remarkably increased in WUD-treated HT-29 cells and HCT116 cells in a concentration-dependent manner (Figures 2(a), 2(b), 2(d), and 2(e)). To verify whether the growth inhibitory effect of WUD was related to the elevation of ROS level, HT-29 cells and HCT116 cells were pre-treated with NAC for 1 h and then treated with WUD for 24 h. As exhibited in Figures 2(c) and 2(f), NAC pretreatment markedly suppressed the growth inhibitory effect of WUD against HT-29 and HCT116 in a dose-dependent manner.

### 3.3. WUD Induces G_0_/G_1_ Phase Arrest in CRC Cells and Suppresses the Expression of Cyclin D1

**Figure 3:**
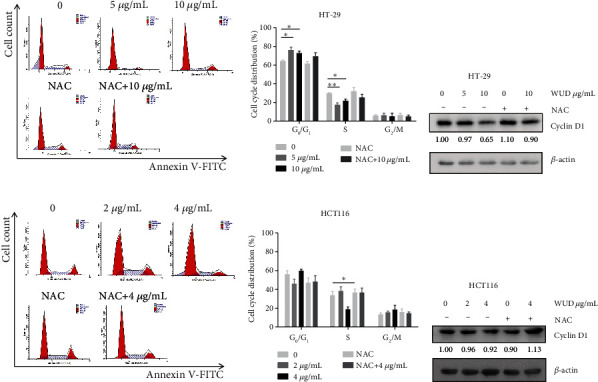
Effect of WUD on cell cycling of CRC cells. HT-29 (a, b) and HCT116 cells (d, e) were treated with WUD for 24 h, then the cells were stained with PI followed by flow cytometry analysis. (c, f) Protein levels of Cyclin D 1 were assessed with western blot after incubated with WUD for 24 h. GAPDH was used as a loading control. Data were mean ± SD. ^∗^*P* < 0.05;  ^∗∗^*P* < 0.01.

To identify whether the growth inhibitory effect of WUD was associated with cell cycle arrest, cell cycle phase distribution of HT-29 cells and HCT116 cells was measured by PI staining and flow cytometry analysis. As depicted in Figures 3(a) and 3(b), treatment of HT-29 cells with WUD at 0, 5, and 10 *μ*g/mL for 24 h induced the accumulation of HT-29 cells in the G_0_/G_1_ phase (76.12 ± 4.20%,  72.87 ± 2.89%, compared to 64.32 ± 1.36% in untreated cells) with a concomitant decrease in the S phase (17.50 ± 2.71%, 21.81 ± 2.59%, compared to 29.83 ± 0.84% in untreated cells). Addition of NAC reversed the WUD-induced G_0_/G_1_ phase arrest in HT-29 cells, which suggests that the increasing ROS induced by WUD was involved in the G_0_/G_1_ phase arrest. Consistently, NAC pretreatment effectively attenuated WUD-induced down-regulation of Cyclin D1, a positive key modulator of the transition through the G_1_/S checkpoint (Figure 3(c)). Collectively, these data suggested that WUD induced cell cycle arrest of HT-29 cells at the G_0_/G_1_ phase and repressed Cyclin D1 expression. In contrast, HCT116 cells performed differently from HT-29 cells. As shown in Figures 3(e), and 3(f), HCT116 cells were treated with WUD at 0, 2, and 4 *μ*g/mL for 24 h, the percentage of cells at G_0_/G_1_ phase was not significantly changed. Similarly, the protein level of Cyclin D1 remained barely unchanged.

### 3.4. Effect of WUD on PI3K, Akt, and GSK3*β* Expression

**Figure 4:**
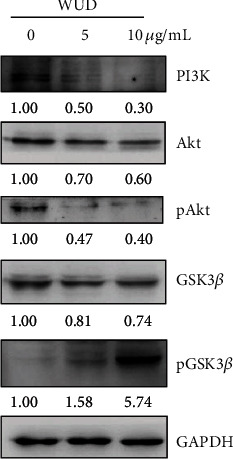
Effect of WUD on PI3K, Akt, and GSK3*β* expression. HT-29 cells were treated with 0, 5, and 10 *μ*g/mL WUD for 24 h; then, the cells were subjected to western blot analysis and detected by PI3K, Akt, p-Akt (Ser473), GSK3*β*, and p-GSK3*β* (Ser9) antibodies. GAPDH was used as a loading control.

The expression of Cyclin D1, a positive key modulator of the transition through the G_1_/S checkpoint, was tightly regulated by the PI3K/Akt/GSK3*β* cascade signaling pathway [30]. To determine whether the PI3K/Akt/GSK3*β* pathway is involved in WUD-induced G_0_/G_1_ phase arrest of HT-29 cells, western blot analysis was employed to evaluate the expression of PI3K, Akt, p-Akt, GSK3*β*, and p-GSK3*β* upon exposure to WUD. As shown in Figure 4, the protein expression of PI3K, Akt, p-Akt, and GSK3*β* was reduced, while the pGSK3*β* protein level was remarkably up-regulated compared with the WUD-untreated control group (Figure 4). These results suggest that the WUD treatment triggered G_0_/G_1_ phase arrest maybe by modulating PI3K/Akt/GSK3*β* signaling pathway.

### 3.5. WUD Induces Apoptosis in CRC Cells

**Figure 5:**
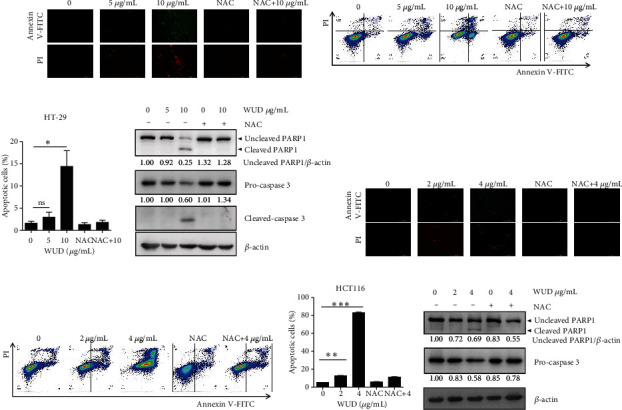
Apoptosis assays in CRC cells. HT-29 (a, b, c) and HCT116 cells (e, f, g) were treated with WUD for 24 h, then the cells were co-stained with Annexin V-FITC/PI and subjected to fluorescence microscopy and flow cytometry analysis. (d, h) Protein levels of uncleaved PARP1, cleaved PARP1, and pro-caspase 3 were assessed with western blot after being incubated with WUD for 24 h. *β*-Actin was used as a loading control. Data were mean ± SD. ^∗^*P* < 0.05;  ^∗∗^*P* < 0.01;  ^∗∗∗^*P* < 0.001. ns: not significant.

To investigate whether the antiproliferative effects induced by WUD were related to apoptosis, an Annexin V-FITC/PI double staining followed by fluorescence microscopy analysis was carried out to determine the distribution of early and late apoptosis after WUD treatment. As shown in Figure 5(a), untreated viable cells exhibited insignificant green and red fluorescence. After being treated with WUD, the number of cells displaying green and red fluorescence significantly increased, indicating early and late apoptosis. The apoptosis of HT-29 cells was confirmed by flow cytometry analysis. As shown in Figures 5(b) and 5(c), when HT-29 cells were treated with WUD at 0, 5, and 10 *μ*g/mL for 24 h, the percentage of apoptotic cells increased from 1.65 ± 0.31% to 2.96 ± 0.80%, 14.39 ± 2.51%, respectively. Also, treatment with WUD resulted in PARP1 and caspase 3 cleavage with a concomitant decrease in pro-caspase 3 protein level (Figure 5(d)). To explore whether the ROS generation participated in the WUD-induced apoptosis, HT-29 cells were cotreated with NAC and WUD. As shown in Figures 5(a)–5(d), pretreatment with NAC effectively ameliorated the WUD-induced apoptosis and reversed the cleavage of PARP1 and caspase 3. Similar results were observed in HCT116 cells (Figures 5(e)–5(h)). Taken together, these results indicated that WUD treatment could activate apoptosis of HT-29 and HCT116 cells.

### 3.6. WUD Inhibits Tumor Growth In Vivo

**Figure 6:**
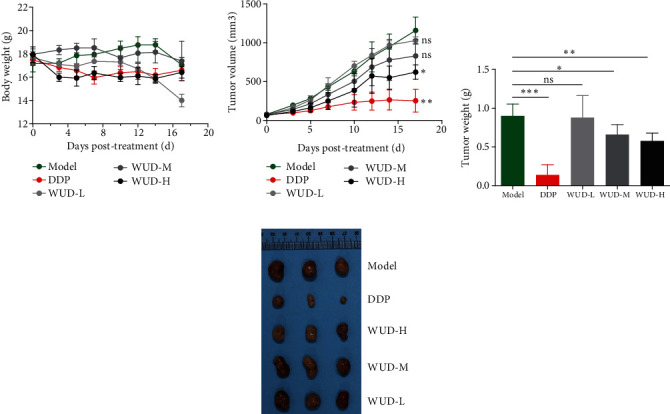
WUD impairs tumor growth in vivo. (a–d) CT26 CRC-bearing xenografted mice were intraperitoneally treated with PBS (model group) or DDP or WUD for 18 days, each with 6 animals/group. (a) Mouse body weights and (b) tumor volumes were measured during the treatment. (c) Tumor weights and (d) the photographs of tumors were measured at the end of the treatment. Data were mean ± SD. ^∗^*P* < 0.05;  ^∗∗^*P* < 0.01. ns: not significant. DDP: cisplatin 5 mg/kg, twice weekly; WUD-L: 57 mg/kg/d; WUD-M: 114 mg/kg/d; WUD-H: 228 mg/kg/d.

To determine the *in vivo* anti-tumor effect of WUD, a Balb/c mouse transplanted tumor model was established. Tumor-bearing mice were treated with indicated concentrations of WUD for 18 days. As shown in Figure 6(a), the weight of the mice were decreased in the WUD-H and WUD-L groups, as well as in the positive control DDP group. Tumor volume was markedly decreased in the WUD-H and DDP groups, but there were no significant changes in the WUD-L and WUD-M group as compared to the model group (Figure 6(b)). At the end of the experiment, a significant tumor weight loss was observed in the WUD-M, WUD-H, and DDP groups. On the contrary, no apparent tumor weight change was found between the WUD-L and model groups (Figures 6(c) and 6(d)).

## 4. Discussion

*W. ugandensis*, widely distributed in Africa, is a traditional herbal medicine used for the treatment of various diseases including cancer. Guéritte et al. reported that the EtOAc extract from the bark of *W. ugandensis* exhibited potent cytotoxic activity against human tumor cell line KB (mouth epidermoid carcinoma) [12]. According to our previous study, WUD improved the intracellular ROS level and then modulated the cell proliferation and G_0_/G_1_ cell cycle progression of A549 cells. Moreover, WUD also exhibited remarkable inhibitory growth activity against HT-29 cells, but the underlying molecular mechanism remained poorly understood [31].

In the present work, we evaluated the anti-CRC activity of WUD and elucidated its possible mechanisms. First, we found that WUD enhanced ROS generation and exerted potent anti-proliferative effect against HT-29 and HCT116 cells in a dose- and time-dependent manner via ROS accumulation. In fact, most of the chemotherapeutic agents used for the treatment of cancers often take their function by inducing apoptosis of cancer cells, which is a major signal pathway leading to cell growth inhibition [32]. Therefore, we employed an Annexin V-FITC/PI double staining and western blotting for apoptosis analysis, and found that WUD treatment activated ROS-dependent apoptosis of HT-29 and HCT116 cells. The most studied apoptosis pathways are caspase-dependent, mainly initiated through death receptor-mediated extrinsic pathway and mitochondria-mediated intrinsic pathway, both of which can be activated by intracellular ROS [33]. As WUD increased intracellular ROS and activated caspase 3-involved apoptosis, both events are closely related to mitochondria, it is reasonable to speculate that treatment of HT-29 and HCT116 cells leads to the dysfunction of mitochondria, resulting in the ROS generation and subsequent apoptosis activation.

In this study, we demonstrated that WUD dramatically increased the intracellular ROS level of HT-29 and HCT116 cells in a dose-dependent manner, which could be reversed by NAC. Notably, the NAC pretreatment also reversed the WUD-induced anti-proliferation and apoptosis in HT-29 and HCT116 cells, and alleviated the G_0_/G_1_ arrest upon WUD treatment in HT-29 cells. Compared with normal cells, cancer cells usually contain a higher basal level of ROS and a highly activated antioxidant defense system to maintain cellular redox homeostasis [34]. Hence, some ROS-inducing anticancer agents cause prominent DNA damage, MMP disruption, and eventually cell death by the accumulation of excessive ROS [29]. Nuclear factor erythroid 2-related factor 2 (Nrf2) is the master regulator of the antioxidant defense system [35]. PI3K/Akt inactivates GSK3*β*, which functions as a negative regulator of Nrf2 by inducing Nrf2 degradation [35]. Therefore, we hypothesized that WUD treatment might impede the antioxidant defense system by inactivating PI3K/Akt/GSK3*β*/Nrf2 pathway, resulting in the increased ROS level.

The PI3K/Akt pathway, one of the most frequently activated signaling pathways in human cancers, functions in pro-survival and anti-apoptosis in cancer [36]. The PI3K/Akt pathway induces tumorigenesis by modulating downstream targets which participated in cell survival and cell proliferation. More importantly, ROS can repress the activation of the PI3K/AKT pathway in pancreatic cancer cells and human leukemia cells [37]. As shown in Figures 2 and 4, we observed that WUD treatment enhanced ROS generation and suppressed the PI3K/Akt pathway as determined by the down-regulation of PI3K, total-Akt, and p-Akt. Multiple studies support the role of Akt in apoptosis suppression and cell cycle promotion by regulating downstream targets, such as BAD, caspase 9, and FoxO for apoptosis and P21, P27, Cyclin D1 for cell cycle progression. Akt phosphorylates BAD on S136 and S112 and creates a binding site for 14-3-3, which triggers BAD release Bcl-2/Bcl-xl, and then free Bcl-2/Bcl-xl will be able to exert an antiapoptotic effect. Cardone et al. reported that Akt directly phosphorylates caspase 9 on S196, resulting in a decrease in the protease activity of caspase 9 [38]. Akt also phosphorylates transcription factor FoxO, triggering FoxO translocates from the nucleus to cytoplasm and abrogates the expression of downstream pro-apoptotic proteins [39]. Akt phosphorylates the p27 and p21, which leads to the export of p27 and p21 to the cytoplasm and attenuates the cell cycle inhibitory effects [40]. Akt activates Cyclin D1 by negatively regulating GSK3*β* and thus facilitates cell cycle progression [30]. Therefore, the Akt pathway may be involved in WUD-induced apoptosis and G_0_/G_1_ arrest in HT-29 cells. Given the central role of Akt in cellular signaling transduction, it is worth noting that other downstream targets of Akt may also contribute to the anti-proliferation effect of CRC cells. Apart from the PI3K/Akt pathway, some ROS-inducing anticancer agents, such as phenethylisothiocyanate (PEITC), benzyl isothiocyanate (BITC), penfluridol acted via downregulating specificity protein (Sp) transcription factors (TFs) Sp1, Sp3, and Sp4, and the downstream EGFR, VEGF, VEGFR1, survivin, Cyclin D1, c-Met, Bcl-2, STAT3, *α*6-integrin, *β*4-integrin, *α*5-integrin, and *β*1-integrin [28, 41–43].

## 5. Conclusions

As an important traditional herbal medicine, *W. ugandensis* has been used in Africa for a long time. There are numerous reports on the anti-bacterial, and anti-fungal activities of *W. ugandensis*, but its anticancer activity is rarely studied, let alone the mechanism of action. In this work, we first found that WUD inhibited the growth of CRC *in vitro* and *in vivo*, and further explored that apoptosis, G_0_/G_1_ phase arrest, and ROS actively participated in WUD-induced antiproliferative effects on HT-29 cells.

## Data Availability

All data is maintained within the manuscript. Raw data is available on request.
